# Metastasis infiltrating tumor to meningioma: a case report

**DOI:** 10.1186/s13256-024-04349-x

**Published:** 2024-02-02

**Authors:** Ryo Matsuzaki, Yutaka Fuchinoue, Masataka Mikai, Chie Nakada, Kei Uchino, Sayaka Terazono, Masashi Harada, Kosuke Kondo, Naoyuki Harada, Nobuo Sugo

**Affiliations:** Department of Neurosurgery (Omori), School of Medicine, Faculty of Medicine, Toho University, 6-11-1, Omori-Nishi, Ota-Ku, Tokyo, 143-8541 Japan

**Keywords:** Metastasis infiltrating tumor, Tumor-to-tumor metastasis, Meningioma, Metastatic brain tumor

## Abstract

**Background:**

There have been many reports of tumor-to-tumor metastasis, in which cancer metastasizes directly into meningiomas. However, metastasis infiltrating tumors in which cancer metastasizes around meningiomas are rare. Therefore, we report a case of metastasis originating from lung cancer that infiltrated meningioma.

**Case presentation:**

A 79-year-old Japanese woman underwent head magnetic resonance imaging for brain metastasis screening before lung cancer surgery. At that time, asymptomatic meningioma of the left frontal region was accidentally found. Magnetic resonance imaging 6 months later revealed a lesion suspected to be a metastatic brain tumor close to the meningioma. Brain tumor resection was performed, and histopathological diagnosis was meningioma and metastatic brain tumor. Metastatic cancer had invaded the meningioma at the boundary between the brain tumor and metastasis.

**Conclusions:**

A sudden change in imaging findings on routine examination of meningiomas in patients with lung carcinoma may indicate a metastatic brain tumor. The form of cancer metastasis to meningioma is not limited to tumor-to-tumor metastasis, but also includes metastasis infiltrating tumors near the meningioma.

## Background

Since the report by Fried of a case of lung cancer metastasizing to meningioma in 1930, there have been many similar reports [1]. The most common primary site of metastasis is breast cancer, followed by lung cancer and kidney cancer [2, 3].

Meningiomas are the most likely to metastasize [4], and the mechanism has been suggested to be hemodynamic, metabolic, and hormonal [5, 6]. There are two forms of cancer metastasis associated with existing tumors: tumor-to-tumor metastasis and metastasis infiltrating tumor. Tumor-to-tumor metastasis is the histological presence of a metastatic brain tumor within an existing primary brain tumor [7], and many case reports describe this form. On the other hand, a metastasis infiltrating tumor is a condition in which a malignant tumor has metastasized to tissues adjacent to an existing primary brain tumor [8], and there are very few reports of them [9–11]. Therefore, we report a case of meningioma with a metastasis infiltrating tumor originating from lung cancer.

## Case presentation

**Fig. 1:**
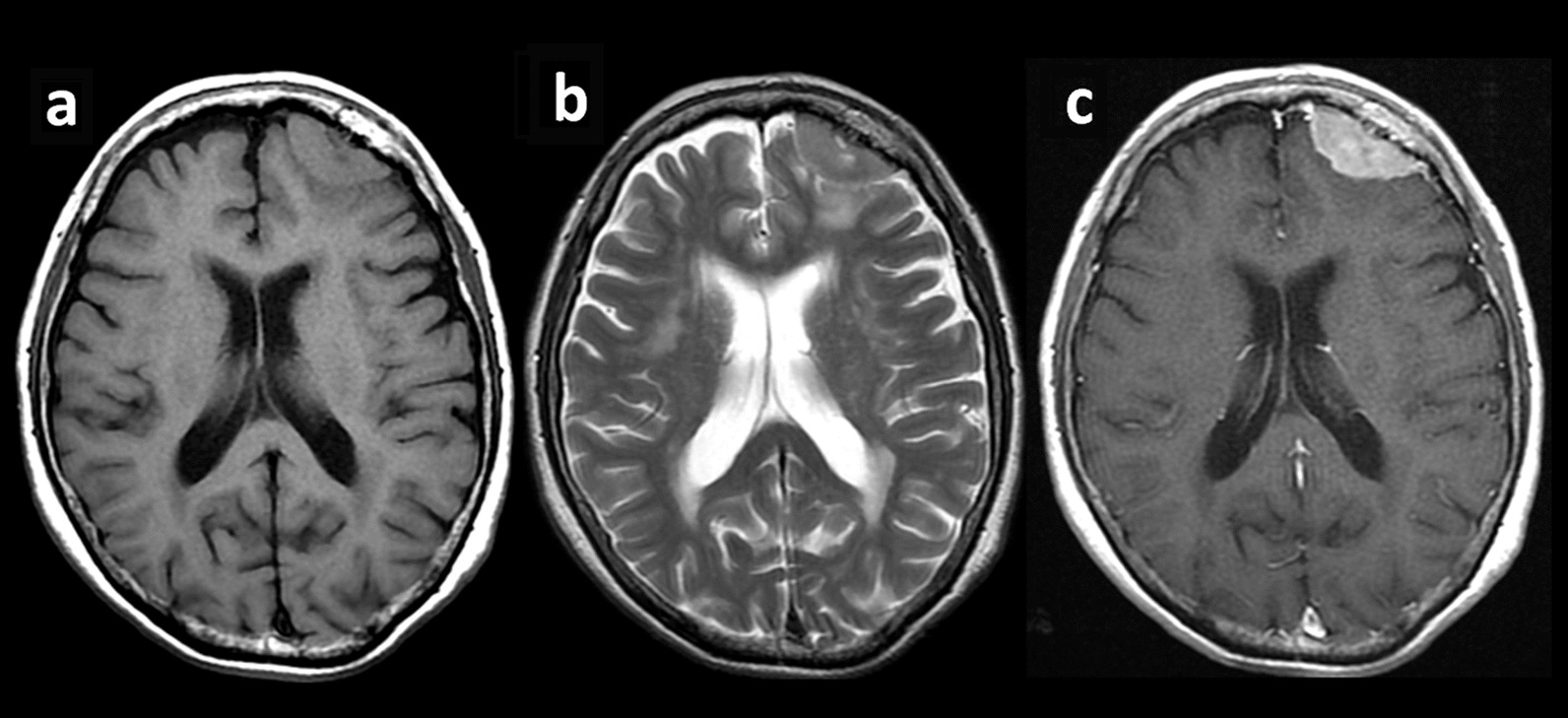
Preoperative magnetic resonance imaging shows an extra-axial mass in the left frontal lesion. Post-contrast T1 weighted images show an enhancing dural-based mass. **a** The axial T1-weighted image. **b** The axial T2-weighted image. **c** The axial contrast-enhanced image

**Fig. 2:**
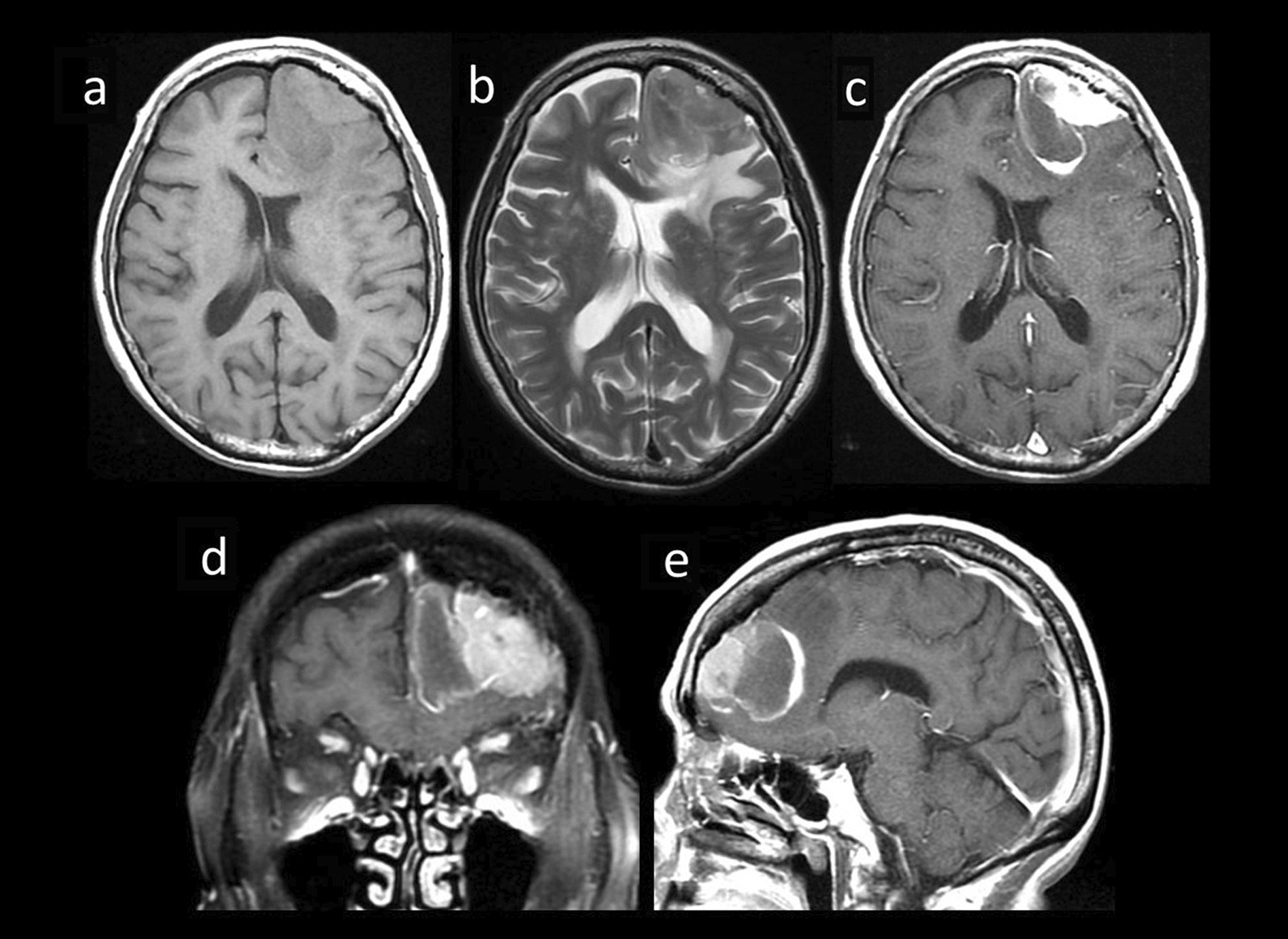
Magnetic resonance imaging 6 months later shows ring enhanced mass by the dural-based mass. **a** The axial T1-weighted image. **b** The axial T2-weighted image. **c** The axial contrast-enhanced image. **d** The coronal contrast-enhanced image. **e** The sagittal contrast-enhanced image

**Fig. 3:**
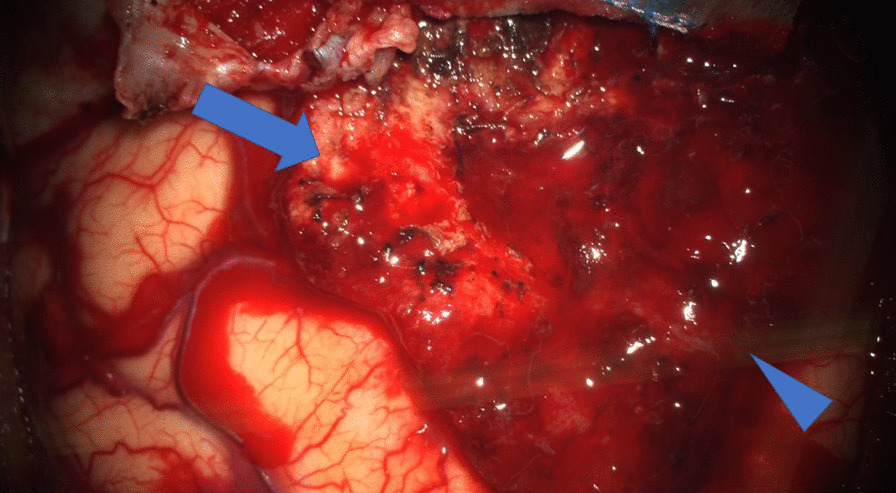
Intraoperative photograph. The upper part is meningioma (arrow), and the lower part is a metastatic brain tumor (arrowhead)

**Fig. 4:**
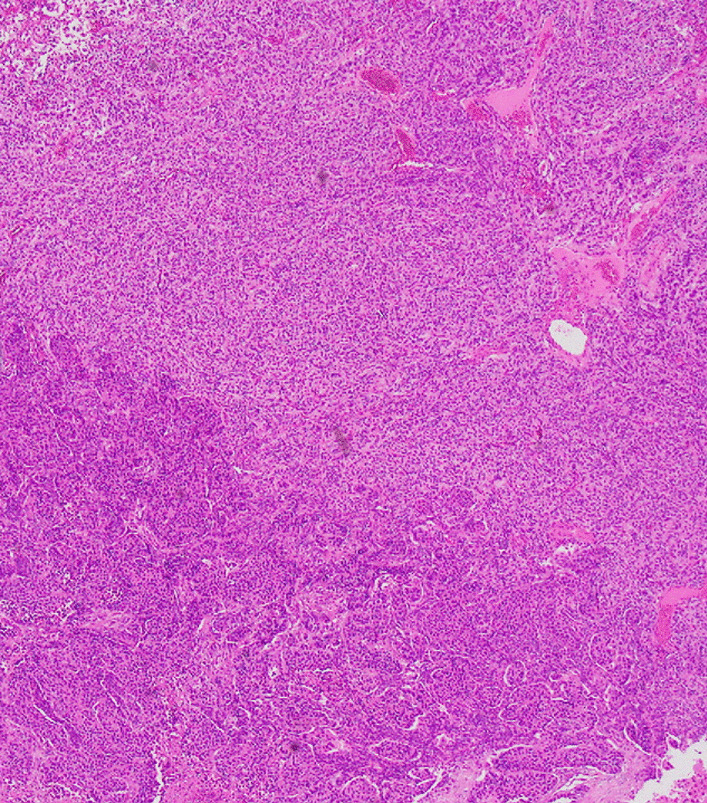
Microscopic images. The upper part is meningioma, and the lower part is a metastatic brain tumor. They are demarcated from one another

**Fig. 5:**
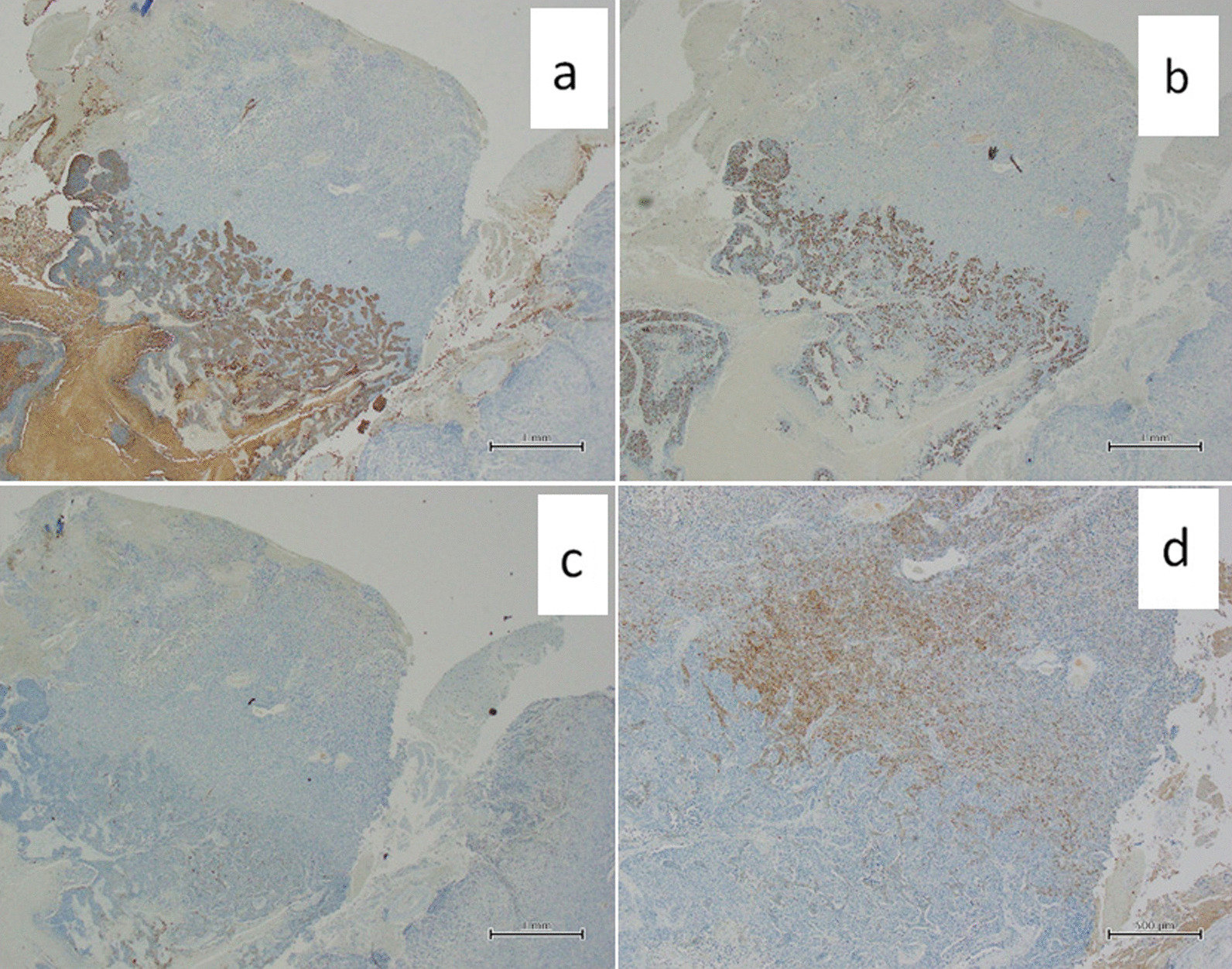
Immunohistochemical examination of cytokeratin (**a**), MIB-1 (**b**), epithelial membrane antigen (**c**), and S-100 (**d**)

This case report describes a case of a 79-year-old Japanese female who underwent thoracoscopic upper left lobectomy with lymph node dissection (pT2aN1M0, stage IIB) for left upper lobe lung cancer. Before the lung cancer surgery, head magnetic resonance imaging (MRI) was performed to screen for brain metastasis. An incidental 37 mm × 16 mm extraparenchymal mass was found in the left frontal region, which was diagnosed as convexity meningioma on imaging (Fig. 1). There were no intracranial metastases. Follow-up MRI 6 months later identified a lesion (46 mm × 28 mm) showing ring enhancement with peritumoral edema adjacent to the left frontal convexity meningioma (Fig. 2). A metastatic brain tumor was suspected, and the patient was admitted to our department for surgery. The patient was alert and had no neurological deficit preoperatively. On chest computed tomography (CT), there was no local recurrence of cancer in the left upper lobe. However, a 15-mm nodule was found in the right lung, suggesting metastasis. The surgery was performed with a frontotemporal craniotomy under general anesthesia. When the dura was inverted, a meningioma was found attached to it, and a metastatic brain tumor with a cystic component was found adjacent to the meningioma. They were removed *en bloc*, including the dura (Fig. 3). The postoperative period was uneventful, and the patient was discharged on the 13th postoperative day. No radiochemotherapy was given because the patient refused. Pathological findings were that the site of the meningioma consisted of cells with round nuclei and weakly acidophilic or pale vacuoles, forming a substantial vesicle with intervening small blood vessels. Additionally, calcification and whorl were observed (Fig. 4). Immunohistochemical staining showed partial positivity for S-100 and negativity for cytokeratin (CK), epithelial membrane antigen (EMA), glial fibrillary acidic protein (GFAP), and p40. The MIB-1 index was less than 1% (Fig. 5). Pathologically, it was diagnosed as World Health Organization (WHO) grade I meningioma. In metastatic brain tumors, atypical cells with enlarged nuclei and basophilic sporangia formed irregular foci, which in this case were accompanied by necrosis. Immunohistochemical staining was positive for p40 and CK and negative for EMA, GFAP, and S-100. The pathological findings were in accordance with previous lung cancer findings, and the MIB-1 index was 65% (Fig. 5). At the boundary between the two tumors, a metastatic brain tumor partially infiltrated into the meningioma tissue (Fig. 4).

## Discussion

The definition of systemic tumor-to-tumor metastasis includes the following: (1) at least two primary tumors must exist; (2) the host tumor must be a true neoplasm; (3) the metastatic focus must show established growth inside of the host tumor and must not be the result of contiguous growth, a collision process, or embolization; and (4) the host tumor cannot be a lymph node involved by leukemia or lymphoma [7]. In the case of intracranial diseases, there have been more than 90 reports of cancer metastasis to primary brain tumors [2, 4, 5, 12–34]. Reasons for the increase in reporting may include increased awareness of the condition, detailed pathologic evaluation, and longer survival for extracranial malignancies [14]. The most common form of extracranial cancer metastasis to existing brain tumors is tumor-to-tumor metastasis, while a rare form is a metastasis infiltrating tumor [8].

Caroli *et al.* reviewed 63 cases of tumor-to-tumor metastasis and found that the most common primary site was breast cancer (27 cases), followed by lung cancer [4]. Since Caroli’s report, 31 cases have been described, including 9 males and 22 females with an average age of 62.4 years, with lung cancer being the most common primary lesion, unlike in previous reports [2, 4, 5, 12–34]. From these cases, all but one found by autopsy were surgically resected, 15 received postoperative radiochemotherapy, and the average survival time after craniotomy was 10.4 months [2, 4, 5, 12–34].

The mechanism of metastasis of extracranial cancer to meningioma as tumor-to-tumor metastasis is speculated as follows: (a) meningioma is a slow-growing tumor, which provides a long period for the metastasis; (b) meningioma is a richly vascularized tumor, which increases the chances of receiving hematogenous metastasis; (c) there is an important association between these two tumors by hormonal influences; (d) the lack of immune response by meningioma favors metastasis development and installation; and (e) the amount of collagen and lipids within meningioma benefits breast cancer metastatic cells [25]. In addition to this, a metastasis infiltrating tumor is thought to involve similar factors as a subtype of tumor-to-tumor metastasis. On the other hand, the mechanism is different in that cancer metastasizes near the existing brain tumor itself [11, 35] and later fuses with it [8]. In this case, there were pathological findings of lung cancer invasion into the meningioma, which occurred in the vicinity of the meningioma. The reason for metastasis near the meningioma is that vascularization is higher in the surrounding tissue than in the tumor [11]. In this case, preoperative contrast-enhanced MRI showed a dural tail sign, suggesting the presence of abundant blood flow around the meningioma. In pathological studies, the dural tail sign has been associated with the presence of numerous small vessels [36, 37]. The edema around the meningioma also suggests the presence of abundant blood flow from the pial vessel [38]. Increased blood flow in the surrounding brain parenchyma associated with the edema may promote metastasis near the meningioma [10].

Neuroradiological imaging can easily diagnose malignant tumor metastasis when the meningioma grows rapidly, as in this case. However, previous reports have shown that diagnosis is often difficult with general imaging tests alone [2, 12]. Perfusion MRIs can non-invasively capture differences in vascularity within a tumor [12]. Vascular-rich meningiomas show high regional cerebral blood flow whereas adenocarcinomas with high tissue mucin content show low regional cerebral blood flow [12]. Magnetic resonance (MR) spectroscopy, which measures metabolites in tissues, is useful for differential diagnosis because metastatic brain tumors and meningiomas have different lipid/creatinine and alanine/creatinine ratios, and metastatic brain tumors have a higher lactate/creatinine ratio [12].

## Conclusion

A sudden change in imaging findings on routine examination of meningiomas in patients with carcinoma may indicate a metastatic brain tumor. In addition to the common tumor-to-tumor metastasis, metastasis infiltrating tumor that occurs in the vicinity of the meningioma should also be considered. Cases of metastasis infiltrating tumor are extremely rare, and knowledge of their existence can be helpful in perioperative management.

## Data Availability

Not applicable.

## Abbreviations

CT: Computed tomography
CK: Cytokeratin
EMA: Epithelial membrane antigen
GFAP: Glial fibrillary acidic protein
MR: Magnetic resonance
MRI: Magnetic resonance imaging
WHO: World Health Organization
